# Assessing Freshwater Microbiomes from Different Storage Sources in the Caribbean Using DNA Metabarcoding

**DOI:** 10.3390/microorganisms11122945

**Published:** 2023-12-08

**Authors:** Joseph Cross, Prasanna Honnavar, Xegfred Lou T. Quidet, Travis Butler, Aparna Shivaprasad, Linroy Christian

**Affiliations:** 1 Department of Biochemistry, Cell Biology and Genetics, American University of Antigua College of Medicine, St. Johns 1451, Antigua and Barbuda; jcross@auamed.net; 2 Department of Microbial Pathogenesis and Immunology, Texas A&M University School of Medicine, College Station, TX 77843, USA; 3 Department of Microbiology and Immunology, American University of Antigua College of Medicine, St. Johns 1451, Antigua and Barbuda; ashivaprasad@auamed.net; 4 Basic Medical Sciences, American University of Antigua College of Medicine, St. Johns 1451, Antigua and Barbuda; xegfredlouq@auamed.net (X.L.T.Q.); travisb@auamed.net (T.B.); 5 Department of Analytical Services, St. Johns 1451, Antigua and Barbuda; linroy.christian@ab.gov.ag

**Keywords:** NGS, metabarcoding, water, microbiome

## Abstract

Next-generation sequencing (NGS) and the technique of DNA metabarcoding have provided more efficient and comprehensive options for testing water quality compared to traditional methods. Recent studies have shown the efficacy of DNA metabarcoding in characterizing the bacterial microbiomes of varied sources of drinking water, including rivers, reservoirs, wells, tanks, and lakes. We asked whether DNA metabarcoding could be used to characterize the microbiome of different private sources of stored freshwater on the Caribbean Island nation of Antigua and Barbuda. Two replicate water samples were obtained from three different private residential sources in Antigua: a well, an above-ground tank, and a cistern. The bacterial microbiomes of different freshwater sources were assessed using 16S rRNA metabarcoding. We measured both alpha diversity (species diversity within a sample) and beta diversity (species diversity across samples) and conducted a taxonomic analysis. We also looked for the presence of potentially pathogenic species. Major differences were found in the microbiome composition and relative abundances depending on the water source. A lower alpha diversity was observed in the cistern sample compared to the others, and distinct differences in the microbiome composition and relative abundance were noted between the samples. Notably, pathogenic species, or genera known to harbor such species, were detected in all the samples. We conclude that DNA metabarcoding can provide an effective and comprehensive assessment of drinking water quality and has the potential to identify pathogenic species overlooked using traditional methods. This method also shows promise for tracing the source of disease outbreaks due to waterborne microorganisms. This is the first study from small island countries in the Caribbean where metabarcoding has been applied for assessing freshwater water quality.

## 1. Introduction

Traditional methods of monitoring the safety of drinking water have relied on culture methods, often for a limited number of pathogenic species, including indicator species such as coliforms (e.g., *Escherichia*, *Klebsiella*, *Enterobacter*, *Citrobacter*).

The advent of next-generation sequencing (NGS) has provided new options for monitoring water quality. NGS on platforms such as Illumina allows billions of reads in a single run and the simultaneous sequencing of hundreds of samples.

In studies of microbiomes involving NGS, two broad approaches have been used: metabarcoding, the deep sequencing of a marker gene such as the 16S rRNA gene to identify all species in a microbiome, or metagenomics, which analyzes the complete DNA content of a microbial community and allows the elucidation of gene families in a community and their potential metabolic functions [1].

In terms of monitoring the safety of drinking water, NGS offers a much more comprehensive assessment of the species or gene families found in a sample, compared to traditional methods. It also avoids bias associated with culture methods, which are usually only assessing the presence of a limited number of species and may overlook an important group. Additionally, it provides relative abundance data, which are important in assessing the dominant taxa in a sample and determining the risk level associated with the presence of a particular species.

A considerable number of studies have now used NGS approaches to analyze various aspects of water quality. General taxonomic surveys and pathogen detection were the focus of a large proportion of these [2]. Recent studies have reported the use of metagenomics or metabarcoding to investigate seasonal variation of cyanobacteria in drinking water reservoirs [3], the microbiome of tap water and bottled mineral water [4], the presence of pathogenic bacteria in private well water and premises plumbing [5], the microbiome of rainwater tanks [6], and the microbiome of a river used as a source of drinking water [7].

Drinking water on the West Indian Island nation of Antigua and Barbuda is monitored by the Department of Analytical Services, which has, until the present time, conducted investigations using traditional methods. These are primarily culture-based and include the multiple tube fermentation technique and the filtration through membrane technique [8]. These methods have drawbacks as they only detect a limited number of species and are time-consuming.

The island has a history of water shortages caused by droughts and a lack of large catchments. Many private dwellings have their own water harvesting systems, which include underground wells and cisterns and above-ground rainwater tanks. The Antigua and Barbuda government has mandated that every new private dwelling constructed must contain a water harvesting system. Commercial entities such as hotels rely on private desalination systems for potable water to supplement municipal water. Water treatment by the average private householder using chlorination is encouraged, but not routinely undertaken. It is, therefore, imperative that we understand possible differences in risks posed by putative microbiome differences between sources.

This pilot study looked at the feasibility of using metabarcoding to characterize the bacterial microbiome of private sources of stored freshwater in Antigua and Barbuda. We asked whether NGS could be used to detect the presence and abundance of bacterial microbiomes in freshwater sources more efficiently and in more detail than culture methods and whether NGS could be used to detect pathogens which are not generally tested for using traditional means.

We hypothesized the following:

1. The bacterial microbiome of different sources would be significantly different in terms of presence and abundance of different taxa;
2. NGS would be more efficient than traditional methods by being less labor-intensive and more comprehensive;
3. Pathogenic bacterial species that are typically not tested for during routine surveillance would be detected using metabarcoding.

## 2. Materials and Methods

This study involved collecting water samples from three distinct sources within a single geographic district, all situated within a 5-mile radius and sharing similar geographic characteristics. These sources included two residential harvested water catchments (a tank and a cistern) and a private well. Notably, the well, equipped with a pump and a tap, diverges slightly from the typical residential category and is classified as a natural water sample and is used only in emergency situations. Conversely, the tank and the cistern, both rain-fed, are piped directly to homes, serving as the primary water source for the residents.

For the collection process, duplicate 50 mL samples were obtained from each source. These samples were gathered in sterile polypropylene containers following the standardized protocol outlined in the American Public Health Association (APHA) guidelines [9]. The samples were taken directly from a tap following surface disinfection, and the water was allowed to run for 2 min to ensure that the sample represented the water from the source and not water from within the line/piping.

During transportation, the samples were maintained at ambient temperature and were shielded from direct sunlight. To ensure sample integrity, they were cooled using gel packs and stored in insulated containers at 4 °C. The samples reached the laboratory within an hour of collection, ensuring minimal degradation or contamination.

The samples were vacuum-filtered through sterile 0.2 µm filters (Merck, Burlington, MA, USA). The gDNA was extracted from the filters using a PowerWater water kit (Qiagen, Germantown, MD, USA) as per the manufacturer’s instructions.

A 16S amplicon PCR, genomic library preparation [V3–V4 region of 16S rDNA (ca. 420 bp)], and Illumina sequencing were conducted by a commercial sequencing facility (GENEWIZ, LLC. South Plainfield, NJ, USA) (Illumina Inc., San Diego, CA, USA, 2020).

A total of 375,940 2 × 150 bp reads were collected. The raw sequencing data were stored in the CyVerse Discovery Environment cloud cyberinfrastructure (https://cyverse.org, accessed on 22 December 2022).

A sequence analysis was conducted using a standard Qiime (version 2 2017.4) analysis integrated into the DNA Subway Purple Line, as previously described (https://dnasubway.cyverse.org/, accessed on 22 December 2022) [10]. A random subsample of 1000 reads were chosen for quality control. The quality control of the sequence data was achieved by trimming sequence reads based on a plot of average quality (Phred score) and base position (https://learning.cyverse.org/dna_subway_guide/#dna-subway-purple-line-metadata-and-qc, accessed on 22 December 2022). Poor base reads are displayed in red in the DNA Subway plot. The base position where poor reads began was noted, and the sequences at or downstream of that position (250 for both forward and reverse reads in this case) were removed from the 3′ end of all reads in the next step in the process, the Divisive Amplicon Denoising Algorithm 2 (DADA2) [11]. At the DADA2 step, erroneous and chimeric sequences were also removed and forward and reverse reads were paired to create the best representation of the sequences in the samples.

The taxa diversity (both abundance and evenness) in the samples were estimated using the Shannon index. To compare the beta diversity among the water sources, a Bray–Curtis similarity matrix was used. The taxonomic diversity was determined using bar plots at the phylum and class levels. Finally, we analyzed those microorganisms which could be identified at the species level, and those with more than 2% abundance were searched in a Risk Group database for potential human and other animal pathogens (American Biological Safety association, Database 2020. https://my.absa.org/tiki-index.php?page=Riskgroups, accessed on 22 December 2022).

## 3. Results

There was a total of 375,940 NGS paired-end reads returned (Table 1).

Following the removal of poor-quality sequences using DADA2, the average read quality was above 25 for the 25th percentile, in line with the guideline recommended by Cyverse (average phred score versus base position).

Alpha rarefication showed that the majority of the taxa present in the samples were detected with a sequencing depth of 1000.

The DNA Subway aligned query sequences with a reference database of 16S rRNA genes (http://greengenes.lbl.gov, accessed on 22 December 2022) [12,13].

**Figure 1:**
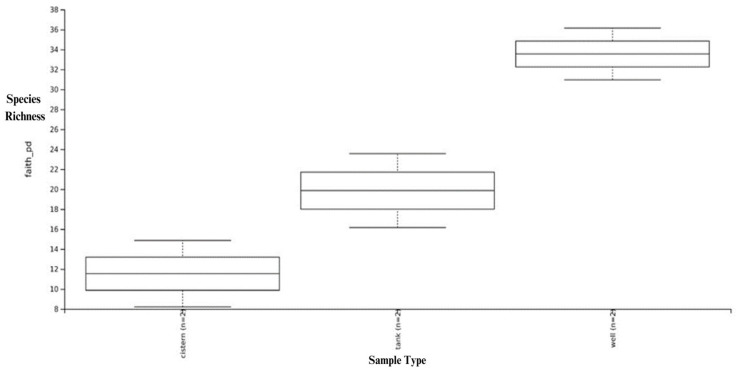
Alpha diversity in the cistern, tank, and well water samples by source.

Alpha diversity provided an estimate of the total diversity in terms of the number of species present in the individual samples. Box and whisker plots for the alpha diversity, as measured using Faith’s phylogenic diversity, a qualitative measure of community richness which incorporates phylogenetic relationships between the features, are shown in Figure 1. The well water sample showed the greatest alpha diversity and the cistern sample the least alpha diversity. The richness and diversity were significantly lower for the cistern water in comparison to the tank and well water.

**Figure 2:**
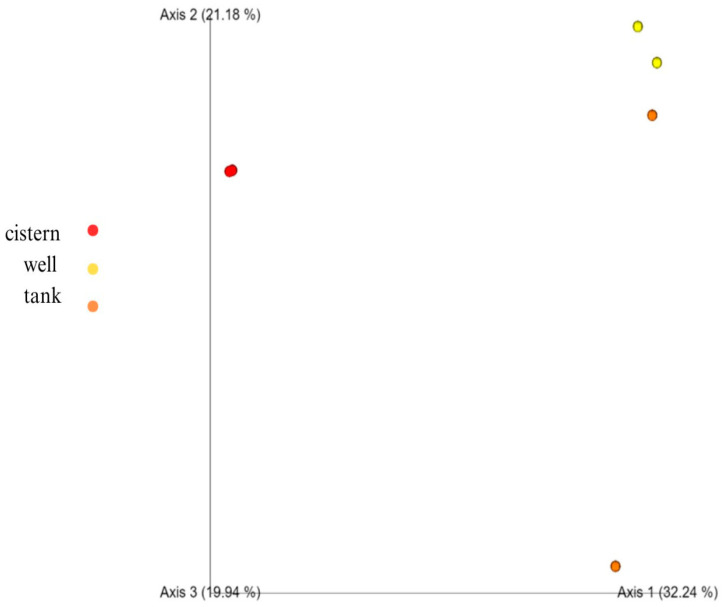
Multi-dimensional scaling (MDS) beta diversity comparison of three water samples.

Bray–Curtis is a metric for describing the dissimilarity of species in ecological sampling. Figure 2 displays a PCoA plot of Bray–Curtis metrics for our samples. Our sample duplicates show distinct clustering by water source. The greatest distance (most dissimilar in taxa composition) was between the cistern samples and all the other samples, with the well and tank water showing the closest relationship. The well samples clustered tightly together, as did the cistern samples, whereas the tank samples did not cluster.

**Figure 3:**
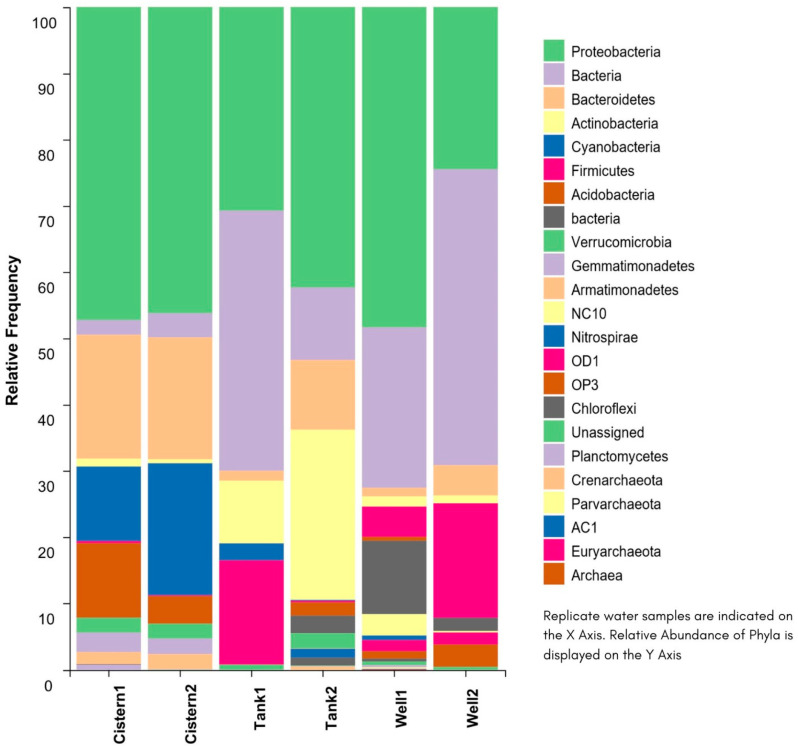
Sample taxonomic diversity based on phylum.

**Figure 4:**
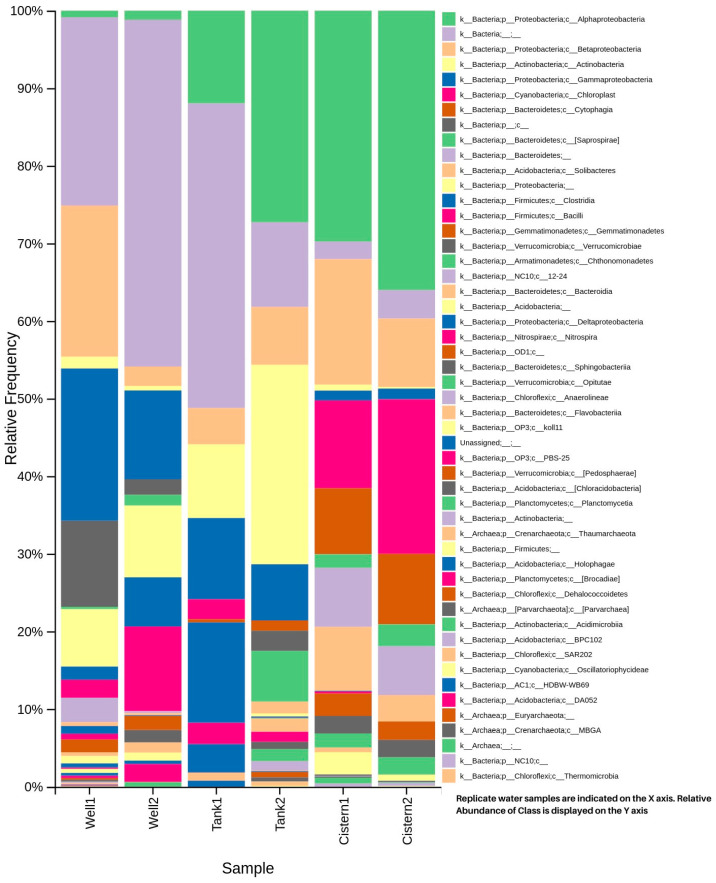
Sample taxonomic diversity based on class.

Figure 3 and Figure 4 display the taxonomic diversity for each sample, based on phylum, and class, respectively. Phyla Proteobacteria was consistently common and abundant among all the freshwater sources (30–48%). Phyla Cyanobacteria (20%), Bacteroidetes (18%), and Acidobacteria (11%) were abundant in the cistern water; Actinobacteria (25%) was abundant in the tank water, and Firmicutes were more common in the tank (15%) and well (17%) water. The class Alphaproteobacteria was more common in the tank (28%) and cistern (36%) water. The class Actinobacteria was more common in the tank water (25%). The class Betaproteobacteria was present in all the samples in abundance (4–20%). The class Gammaproteobacteria was more common in the well (19%) and tank (10%) water. Chloroplast (19%) and cytophagia (9%) were more common in the cistern.

Table 2 lists the taxa and sample source of the human and animal pathogens identified, as well as the characteristics of other species identified at the species level. The human pathogenic bacteria identified at the species level were *Clostridium paraputrificum* from the tank and well samples. Other possibly pathogenic species identified were the *Corynebacterium pilosum* and *Legionella* spp., identified in the tank samples.

## 4. Discussion

Our study explored the bacterial microbiome of different freshwater sources on the West Indian Island of Antigua using 16S rRNA metabarcoding. We found major differences in microbiome composition and abundances related to the water source. Pathogenic species were also detected in all the sources.

Our results demonstrate that alpha diversity, a measure of species diversity, was lowest in the cistern sample. Similarly, beta diversity, a measure of dissimilarity between species based on phylogeny, showed the cistern sample to be most distant to the other samples, although all three samples clearly showed significant differences in microbiome composition and relative abundance.

There are a number of possible reasons for these findings. The cistern our sample was taken from was located at a depth of approximately 6 feet below the residential premises, with no exposure to direct sunlight. Sunlight is known to have a direct impact on freshwater microbiomes through photoinactivation [14]. We are limited in quantitation, but we may note that the number of reads for the cistern samples were generally higher than for the other samples. By the same token, the water from the well was exposed to direct sunlight for part of the day and the above-ground tank was exposed to filtered light through its opaque plastic construction material. We believe that these varying exposure levels contributed to the observed alpha and beta diversity differences.

In addition, we expect the varying levels of sunlight exposure related to the water sources in our study to also have impacted the water temperature. Temperature has previously been shown to impact the bacterial composition of drinking water microbiomes [15]. Future experiments with more comprehensive metadata will be required to confirm this.

Another factor which can impact drinking water microbiomes, including the presence of pathogenic species, is stagnation [16]. The well sampled is only used in emergency situations, such as when government-supplied water is interrupted. This can be compared to the cistern and tank sources, which are the main water sources for the premises, meaning turnover is relatively frequent. Stagnation in the well sample would explain some of the variation seen between the samples.

The taxonomic diversity for the samples showed differences between all three samples. At the phylum level, we detected Proteobacteria and Bacteroidetes at a high relative abundance in our cistern sample. Similar results have been detected in freshwater and domestic sewage sludges from China, Brazil, and Peru [7,17,18,19,20,21]. The abundance of metabolically active Proteobacteria has been related to global nitrogen, carbon, and sulfur cycling [22]. The tank and well samples contained a high level of Firmicutes. Similar results were found in domestic sewage sludges from China and Brazil [17,18,19]. The presence of Cyanobacteria and Acidobacteria in cistern water is unique compared to other freshwater studies. Further studies are warranted to investigate the importance of these phyla.

The classes Actinobacteria and Alphaproteobacteria were common in our tank sample. These were also found to be common in high altitude lakes of Tibet and the Pyrenees [23,24]. The presence of Choloroplast and Cytophagia was unique in our cistern samples.

Significantly, we detected pathogenic species, or genera known to harbor such species, in all the samples.

The presence and high relative abundance of *Legionella* in the tank sample correlates with the results of Hamilton et al., who found *Legionella* spp. to be the most common pathogenic genus in a study of tank water in Australia [25]. The *Legionella* spp. is known to thrive at higher temperatures, which may explain its presence in the tank sample but not in the other samples [26].

The detection of *Clostridium paraputrificum* is alarming, as it has been involved in septicemia and septic arthritis cases in humans [27,28,29,30], especially among immunocompromised patients and individuals with an extreme age range. In addition to the human pathogens, an animal pathogenic species was also detected, *Corynebacterium pilosum* [31,32].

Although we are limited in knowing the absolute quantity of the pathogenic species detected here and, therefore, the risk posed to public health, detection of these species means that further tests, such as qPCR, can now be undertaken to confirm the presence and quantity of each species.

Our results indicate the usefulness of NGS in identifying potential bacterial pathogens in freshwater samples. In particular, our demonstration of the efficacy of metabarcoding for the detection of a wide range of species in a relatively short time period has implications for tracing the source of outbreaks. Currently, multiple potential pathogens must be cultured from suspect samples, which typically does not include all possible pathogens or indicate the relative abundance in a sample. In addition, difficult-to-culture species, or those requiring very specialized media, present a problem when sourcing an outbreak. Metabarcoding, in contrast, will allow detection of all pathogenic species and their relative abundance in a sample and, thus, allow the appropriate intervention to limit the spread of the microorganism and associated illness.

Diseases related to waterborne microorganisms are a significant source of morbidity and mortality in the Caribbean region. It is estimated that 2.1% of the population of Antigua and Barbuda do not have access to safe drinking water and that the percentage is rising (https://www.worldometers.info/water/antigua-and-barbuda-water/, accessed on 16 November 2023). In addition, the Pan American Health Organization (PAHO) has noted that 28 million people in the regions of the Americas (which includes the Caribbean) do not have access to safe water supplies (https://www.paho.org/en/topics/water-and-sanitation, accessed on 16 November 2023). We suggest that metabarcoding provides a valuable new tool for routine water safety surveillance, in addition to the tracing of outbreaks, particularly where resource-sharing between different Caribbean nations would make the process even faster and more cost-effective, as is the case with the COVID-19 NGS laboratory based in Trinidad (https://carpha.org/More/Media/Articles/ArticleID/533/CARPHA-Implements-Whole-Genome-Sequencing-for-Member-States-to-Identify-COVID-19-Variants, accessed on 16 November 2023).

One limitation of NGS for water quality assessment is that fecal indicators and pathogens are frequently at very low abundances [1]. However, our results demonstrate that metabarcoding is capable of detecting species with a low relative abundance, including fecal indicators and pathogenic species, as noted above. Also, the alpha rarefication data suggest that our sequencing depth was sufficient to detect the majority of taxa.

Another anomaly is that tank replicate sample number 1 is quite a long distance in terms of species diversity from tank replicate sample number 2. It has been suggested that the variation between single sample replicates is related to the limitations of membrane filters, such as pore size [33,34]. We note that this replicate for the tank sample had the lowest number of reads and the shortest average read length of all the samples, possibly affecting the detection and abundance data for some species and skewing the results. It has been suggested that increasing biological and PCR replicates will provide more accurate results and allow more valid comparisons between sites and experiments [33,34]. Due to the limited resources available for the project, we elected to sample in duplicate at each location. The duplicate samples provided a clear understanding of the microbial taxa diversity at two of the three sample locations. However, sampling at the well location yielded ambiguous results. Our data demonstrate high alpha diversity in the well samples, though it is unclear if this detected diversity is due to a sampling issue or true sample heterogeneity. Future studies at this location will include collecting an increased number of sample replicates.

Future studies will also link significant differences in species composition between sources to more extensive metadata, such as nitrogen content, temperature, and pH. Staff at the Antigua and Barbuda Analytical Services Department should be trained, and reagents and equipment obtained, to prepare for tracing future disease outbreaks due to waterborne microorganisms. We also suggest that residential and hotel water supplies should be monitored periodically for the presence of pathogens using metabarcoding.

## 5. Conclusions

In conclusion, our results demonstrate that metabarcoding has significant advantages over traditional methods for sanitary water supply outbreak investigations and monitoring. It is both faster and more comprehensive, which enables quicker interventions to control outbreaks. In terms of prevention of waterborne diseases, metabarcoding also has advantages over traditional methods, allowing the detection of a wider range of pathogenic microorganisms during routine monitoring. This is very important for both the country of Antigua and Barbuda and the Caribbean region in general, because of the inadequate supply of safe drinking water in the region.

## Figures and Tables

**Table 1:** Paired-end reads per sample.

Sample	#PE_Reads	#No Chimera	Avg Len (bp)	GC (%)	Effective (%)
Cistern 1	81,597	75,309	427.63	54.23	92.29
Cistern 2	84,702	79,366	437.26	54.39	93.7
Tank 1	23,078	19,020	317.18	56.38	82.42
Tank 2	91,851	86,404	435.12	53.81	94.07
Well 1	69,911	64,347	386.06	50.95	92.04
Well 2	24,801	21,947	367.81	43.36	88.49
Total	375,940				

**Table 2:** Pathogenic taxa detected by source and abundance.

Bacterial Species	Source	Percent-Age Abundance	Comment
*Novosphingobium stygium*	Cistern 1 and 2	2–5	Isolated from deep-terrestrial-subsurface sediments. Degrades aromatic compounds
*Hyphomicrobium sulfonivorans*	Cistern 1, Tank 2	3–4	The bacteria is a producer of enzyme dimethylsulfide monooxygenase. It degrades Dimethylsulfide (DMS). DMS is a volatile organosulfur compound that has been implicated in the biogeochemical cycling of sulfur and in climate control.
*Novosphingobium nitrogenifigens*	Cistern 1 and 2, Tank 2	3–4	Found in pulp and paper wastewater in New Zealand.
*Paracoccus kawasakiensis*	Tank 2	3	Found in an active sludge sample from a food plant in Japan.
*Clostridium paraputrificum*	Well 1 and 2, Tank 1	<3	Pathogenic to humans. Has been isolated from septicemia, septic arthritis, osteomyelitis, liver abscess, and necrotizing enterocolitis cases.
*Novosphingobium acidiphilum*	Cistern 1 and 2	2	Isolated from the subsurface water of the lake Grosse Fuchskuhle in Germany.
*Reyranella massiliensis*	Tank 1 and 2	2	Associated with amoebae.
*Corynebacterium pilosum*	Tank 1	6	Has been associated with human prosthetic valve endocarditis, urinary tract infection (UTI) cystitis, vulvovaginitis in cattle, and UTI in dogs.
*Nitrosospira*	Tank 2	2	Ammonia-oxidizing bacteria from the soil.

## Data Availability

Publicly available datasets were analyzed in this study. These data can be found at https://de.cyverse.org/data/ds/iplant/home/josephcross/analyses?selectedOrder=asc&selectedOrderBy=name&selectedPage=0&selectedRowsPerPage=100, accessed on 22 November 2023.
